# Does ganciclovir exert retinal toxicity after multiple continuous intravitreal injections?

**DOI:** 10.1186/s12879-021-06365-4

**Published:** 2021-07-12

**Authors:** Feng Hu, Ya Ma, Xiaoyan Peng

**Affiliations:** 1 Beijing Institute of Ophthalmology, 17 Hougou Lane, Chongnei Street, Beijing, 100005 People’s Republic of China; 2 Beijing Tongren Eye Center, Beijing Tongren Hospital, Capital Medical University, 17 Hougou Lane, Chongnei Street, Beijing, 100005 People’s Republic of China; 3 Beijing Ophthalmology and Visual Science Key Laboratory, 17 Hougou Lane, Chongnei Street, Beijing, 100005 People’s Republic of China

**Keywords:** Retinal toxicity, Ganciclovir, Intravitreal injection, Acute retinal necrosis, Viral uveitis, Visual field

## Abstract

**Background:**

The objective of this study is to report a case of acute retinal necrosis in which abnormalities in visual function did not correspond to retinal anatomical outcomes.

**Case presentation:**

A 39-year-old female diagnosed with acute retinal necrosis underwent repeated (nine rounds) intravitreal ganciclovir injection (3 mg/0.1 ml) into the left eye, one injection every 2 weeks. During the therapy, the patient noticed her visual acuity declining gradually. The best corrected visual acuity in the left eye was 20/33. The visual field showed massive visual damage. There was no posterior necrotizing involvement, no macular edema or exudation, and only slight abnormity of the interdigitation zone in the fovea area was visible on OCT. Angio-OCT revealed normal capillary density of three retinal capillary and choriocapillaris layers. The visually evoked potential was normal. The photopic single-flash response showed a declined amplitude of a-wave and b-wave. The amplitudes of photopic 30 Hz flicker were decreased. Multifocal electroretinography revealed macular dysfunction.

**Conclusion:**

Ganciclovir-associated photoreceptor damage may induce abnormalities in retinal function in response to multiple continuous intravitreal ganciclovir injections at a relatively high dosage (3 mg/0.1 ml).

## Background

Acute retinal necrosis (ARN) was first described in 1971 by Urayama and colleagues as a syndrome of acute panuveitis with retinal periarteritis progressing to necrotizing retinitis and retinal detachment [1]. Ganciclovir is an antiviral drug developed by Kelvin K Ogilvie in 1982 to treat herpesvirus [1]. Most of the known side effects of intravitreal ganciclovir are related to procedure rather than the drug itself. These include retinal detachment, vitreous hemorrhage, endophthalmitis, subconjunctival hemorrhage, and cataracts [2]. Retinal toxicity was reported to occur in response to high-dose intravitreal ganciclovir (40 mg/0.1 ml and 4 mg/0.04 ml) injection, and patients developed permanent retinal damage and visual loss developed [3, 4]. We report a case of acute retinal necrosis in which the retinal structure appeared relatively normal after repeated doses of intravitreal ganciclovir, but there were abnormalities in visual function, including visual field damage, abnormal electroretinography, and multifocal electroretinography.

## Case presentation

**Fig. 1:**
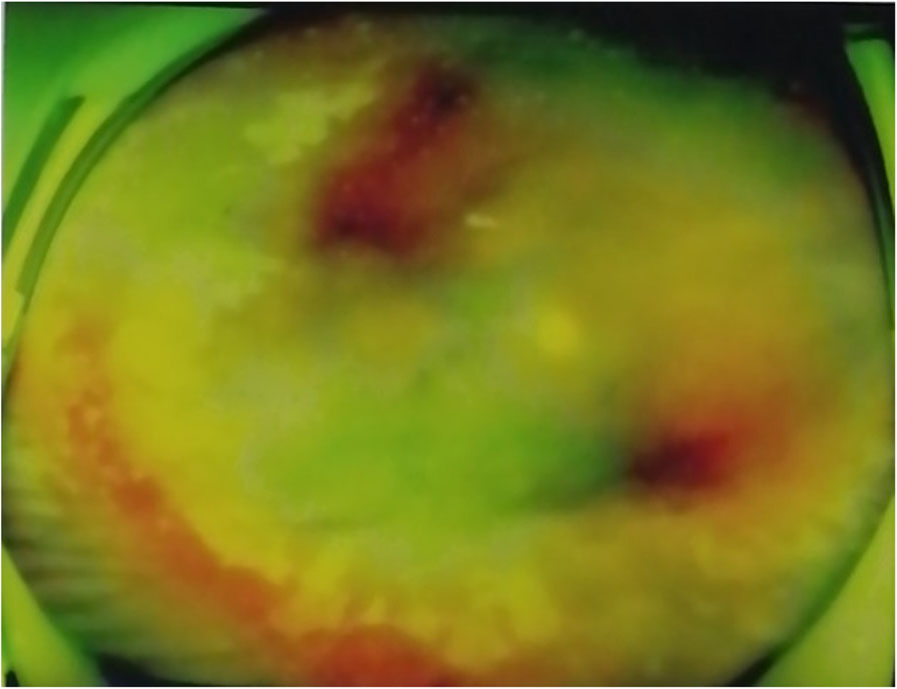
Wide-field photograph of the right eye at initial presentation (five months ago). Wide-field photograph of the right eye showed patchy retinal necrotic lesions in superior and inferior peripheral retina, and diffusive retinal necrotic lesions in the temporal peripheral retina

A 39-year-old female came to our clinic complaining of gradual loss of visual in the left eye for 5 months. She had been diagnosed with acute retinal necrosis in the right eye, and wide-field photograph of the right eye showed patchy and diffusive retinal necrotic lesions (Fig. 1). Pars plana vitrectomy was performed due to retinal detachment of the right eye 5 months earlier. The visual acuity in the left eye at the time of initial presentation was 20/20. In the meantime, she noticed floaters in her left eye. Superior retinal necrosis had been detected and it was recorded in her previous medical chart. The aqueous fluid from the left eye was positive for herpes zoster virus (VZV). She received repeated (nine rounds) intravitreal ganciclovir injection (3 mg/0.1 ml) into her left eye, one injection every 2 weeks, and systemic antiviral therapy for 5 months. The patient noticed a decrease in the visual acuity of the left eye and was referred to our clinic.

**Fig. 2:**
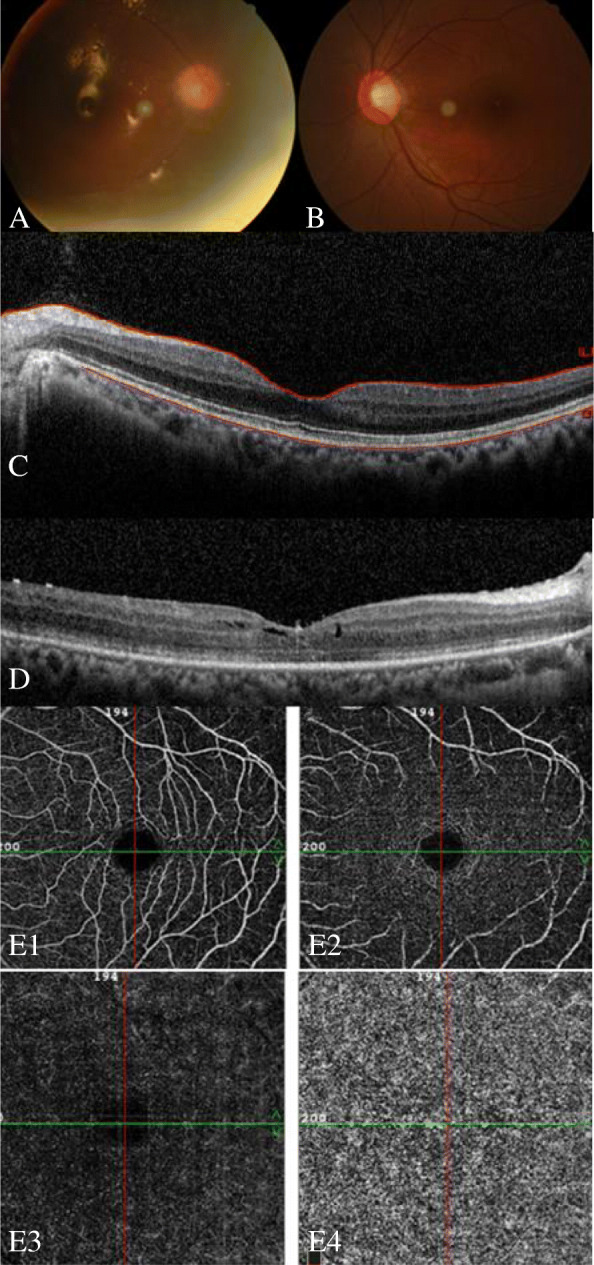
Multi-images of a 39-year-old female acute retinal necrosis patient. **A** The right fundus showed silicone oil tamponade. **B** The left fundus showed no necrosis, no exudation, and no edema in the macular area, and the optic disc appeared normal. **C** Optical coherence tomography of left eye showed slight abnormality of the interdigitation zone in fovea area. **D** Optical coherence tomography of the right eye showed macular edema and rupture of the interdigitation zone. **E**1–4 Superficial retinal capillary, deep retinal capillary, outer retina, and choriocapillaris layers of angio-OCT for the left eye

**Fig. 3:**
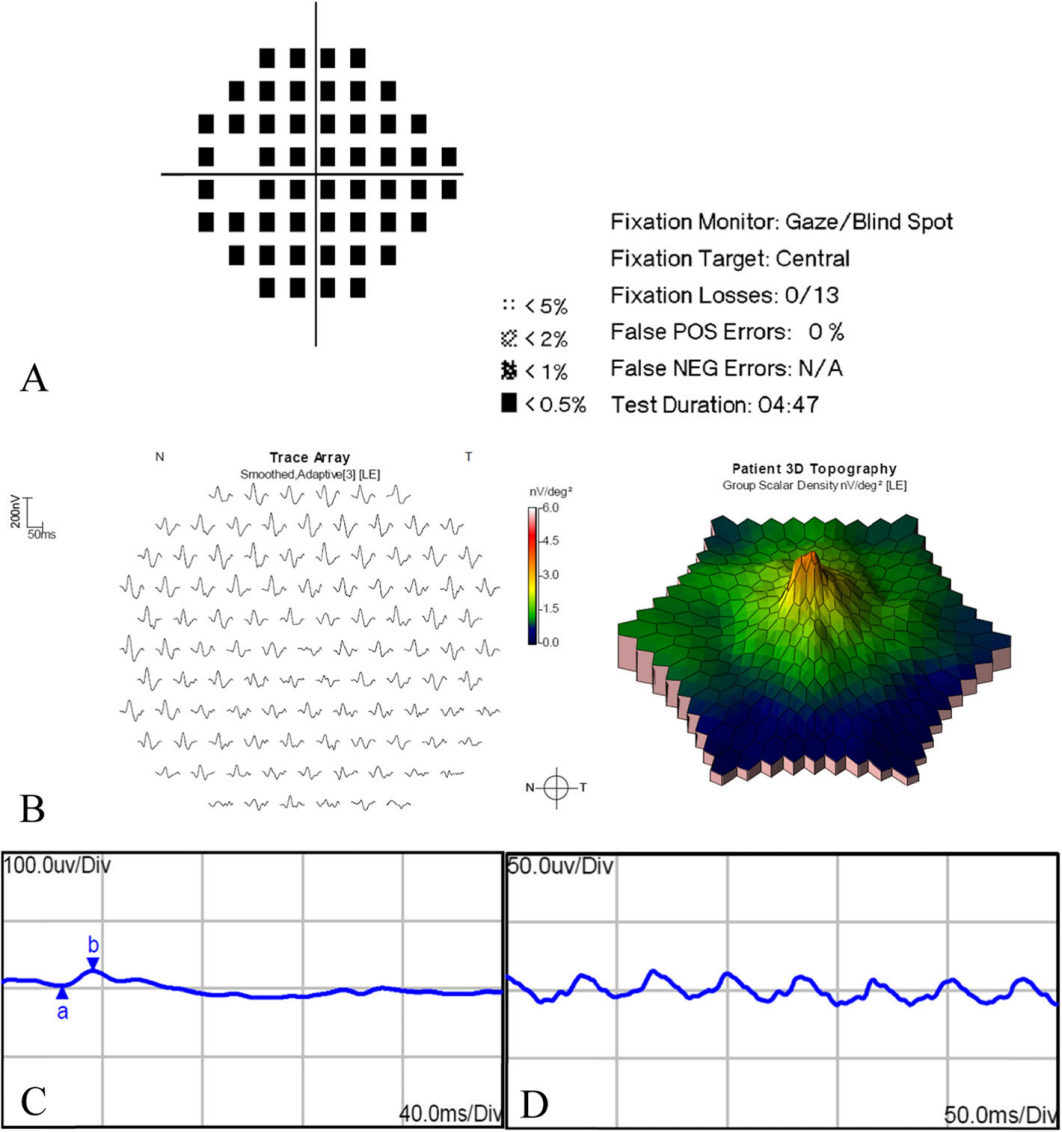
Humphry visual filed, multifocal electroretinography, and electroretinography of the left eye of in a patient with acute retinal necrosis. **A** Humphrey visual field showed massive visual field damage. **B** Trace arrays showed subnormal multifocal electroretinography (mERG) with decreased amplitudes for the left eye. The three-dimensional topographical map showed blunted foveal peak. **C** Photopic single-flash response showed an amplitude of 7.95 uv and interval time of 24 ms for the a-wave, and an amplitude of 21.72 uv and interval time of 36.4 ms for the b-wave. **D** The amplitudes of photopic 30 Hz flicker were 15.73 uv

When the patient came to our clinic, her best corrected visual acuity was 20/400 OS, 20/33 OS. The intraocular pressure was within the normal range in both eyes. There was no inflammation in the anterior chamber, and the lens was normal in the left eye. There was no posterior necrotizing involvement and no macular edema or exudation in the left eye. Indirect ophthalmoscope examination revealed pigmentary change in the superior peripheral retina of the left eye. Slight abnormality of the interdigitation zone in the fovea area of the left eye was detected on OCT. Angio-OCT revealed normal capillary density of superficial retinal capillary, deep retinal capillary, outer retina, and choriocapillaris layers in the left eye (Fig. 2). The Humphrey visual field (central 24–2 threshold test) showed massive visual field damage in the left eye. The amplitude and implicit times of pattern visually evoked potential (VEP) and flash VEP were within the normal range in the left eye. The amplitudes of dark-adapted flash electroretinography (ERG) were slightly low in the left eye. Photopic single-flash response showed decreased amplitude of a-wave and b-wave in the left eye. The amplitudes of photopic 30-Hz flicker were decreased in the left eye. Trace arrays showed subnormal multifocal electroretinography (mERG) with decreased amplitudes for the left eye. The three-dimensional topography map showed a blunted foveal peak (Fig. 3).

## Discussion and conclusions

We here report a case of acute retinal necrosis in which the retinal structure appeared relatively normal after repeated doses of intravitreal ganciclovir, but the visual function abnormalities were remarkable. The posterior segment showed not involvement with retinal necrosis, and there was no macular edema or exudation. Flash and pattern VEP appeared normal for the left eye, so optic nerve involvement of the left eye was excluded. The anatomical outcome was good, with only slight abnormality of interdigitation zone in fovea area in the left eye on OCT. The abnormal ERG revealed retinal dysfunction. The mERG revealed macular dysfunction. What is unusual in the present case is that she accepted nine rounds of continuous intravitreal ganciclovir injections at a relatively high dosage (3 mg/0.1 ml).

Viral infection and inflammation in ARN induce damage to retinal structures and corresponding visual function abnormities. Photoreceptor damage in ARN was here confirmed by OCT observations: hyper-reflective vertical strips within the outer nuclear layer, retinal disruption, and interruption of photoreceptors [5]. Abnormal ERG exams indicating photoreceptor and bipolar cell damage may be associated with ARN induced retinal function damage. What is special in the present case is that abnormal ERG and visual field confirmed abnormal retinal function. In the meantime, the retinal anatomical outcome was relatively good and there were no detectable ARN-induced structural changes.

Intravitreal ganciclovir injection has been used to treat ARN and cytomegalovirus retinitis (CMVR). The recommended dosage of intravitreal ganciclovir used to treat herpetic uveitis is 200–400 μg/0.1 ml. Intravitreal ganciclovir injection at doses of 1 mg, 2 mg, 3 mg and 5 mg has also been used to treat ARN and CMVR [2, 6–8]. In one prospective interventional case series, intravitreal ganciclovir injection of 5 mg/0.1 ml once a week was used to treat active CMVR, and no retinal toxicity was observed [2]. The dosage of intravitreal ganciclovir injection capable of causing retinal toxicity is still unclear. Intravitreal ganciclovir (at doses of 40 mg/0.1 ml, and 4 mg/0.04 ml) has been reported to show retinal toxicity in previous works, with sudden formation of precipitated crystalline material after the intravitreal injection accompanied by high intraocular pressure. The decrease in visual acuity occurred immediately after intravitreal injection [3, 4]. Retinal edema, cherry spot, and delayed arm to retina time supported the conclusion that retinal artery occlusion occurs in the acute retinal toxicity of ganciclovir [4]. The alkaline nature of the solution, osmotic damage, and precipitation of ganciclovir within the retina may cause direct or indirect cellular injury and act in etiology of retinal toxic reaction produced by the highly concentrated ganciclovir [3].

In the present case, there was no sudden decline in visual acuity, and the IOP remained within normal range. The retina and optic disc appeared normal. In this way, there was no evidence supporting a diagnosis of acute retinal toxicity of ganciclovir, as in previous examinations of the present patient. In addition to acute intraocular inflammation and retinal arterial occlusion, photoreceptor damage was confirmed in ganciclovir associated retinal toxicity with loosely arranged swollen photoreceptors, and decreased photoreceptor outer segments upon transmission electron microscope examination [9]. A decrease in a-wave amplitude on ERG indicated photoreceptor dysfunction in the present case, which may be associated with ganciclovir toxicity, but it differed from previous reports of acute retinal toxicity. The limitation of this case is that there was no continuous imaging and functional exams during intravitreal ganciclovir injections.

In conclusion, after excluding ARN associated direct structural damage and acute retinal toxicity of high concentrations of ganciclovir, ganciclovir-associated photoreceptor damage was found capable of inducing abnormalities in retinal function after multiple continuous intravitreal ganciclovir injections at a relatively high dosage (3 mg/0.1 ml), which merits further investigation.

## Data Availability

All data of this case report is included in this published article.

## Abbreviations

ARN: Acute retinal necrosis
VZV: Herpes zoster virus
OCT: Optical coherence tomography
VEP: Visually evoked potential
ERG: Electroretinography
CMVR: Cytomegalovirus retinitis
